# Efficacy and Mechanism of the Jiangtang Tiaozhi Recipe in the Management of Type 2 Diabetes and Dyslipidaemia: A Clinical Trial Protocol

**DOI:** 10.3389/fphar.2022.827697

**Published:** 2022-02-04

**Authors:** Haoran Wu, Xinyi Fang, De Jin, Runyu Miao, Jiahua Wei, Tianyu Zhao, Dan Dai, Jiangquan Liao, Jia Wang, Fengmei Lian, Jiaxing Tian

**Affiliations:** ^1^ Department of Endocrinology, Guang’anmen Hospital, China Academy of Chinese Medical Sciences, Beijing, China; ^2^ Graduate College, Beijing University of Chinese Medicine, Beijing, China; ^3^ Graduate College, Changchun University of Chinese Medicine, Changchun, China; ^4^ Department of Endocrinology, First Teaching Hospital of Tianjin University of Traditional Chinese Medicine, Tianjin, China; ^5^ Department of National Integrated Traditional and Western Medicine Centre for Cardiovascular Disease, China-Japan Friendship Hospital, Beijing, China; ^6^ Department of General Medicine, Guang’anmen Hospital, China Academy of Chinese Medical Sciences, Beijing, China

**Keywords:** Jiangtang Tiaozhi recipe, type 2 diabetes, dyslipidaemia, clinical trial protocol, herbal medicine

## Abstract

**Background:** Type 2 diabetes mellitus (T2DM) complicated with dyslipidaemia is associated with a high risk of cardiovascular diseases. The Jiangtang Tiaozhi (JTTZ) recipe is a Chinese herbal formula that has been used to regulate the blood glucose and lipid levels for many years. Interestingly, a previous study has demonstrated its efficacy; however, the associated mechanism remains unclear. We hypothesised that the therapeutic effect of the JTTZ on patients with T2DM may be mediated by the modulation of metabolites secreted by the gut microbiota. This study aims to examine this mechanism.

**Methods and analysis:** This study is a randomised, positive drug parallel-controlled, open-label clinical trial in patients with T2DM and dyslipidaemia. A total of 96 patients will be recruited and randomly assigned to treatment with JTTZ or metformin for 12 weeks. The primary outcome will be the rates of effectively regulated blood glucose and lipid levels (measured with the levels of glycated haemoglobin, fasting plasma glucose, 2-h plasma glucose, triglyceride, and low-density lipoprotein cholesterol). The secondary outcomes will be the changes in body weight, body mass index, and waist circumference and Traditional Chinese Medicine symptom scores. In addition, 16S rRNA gene sequencing will be performed on the gut microbiota obtained from faeces, and metabolomics analysis will be performed based on blood and gut microbiota samples. Intention-to-treat, per-protocol analysis and safety analysis will be performed.

**Clinical trial registration number:** https://clinicaltrials.gov/ct2/show/NCT04623567

## Background

Type 2 diabetes mellitus (T2DM) and dyslipidaemia are associated with an increased cardiovascular disease (CVD) risk. (Wilson et al., 1998) (Gerstein et al., 2005). Although dyslipidaemia is an independent risk factor for CVD, it plays a major role in the context of T2DM-induced CVD. (Eliasson et al., 2011) (Turner et al., 1998). The prevalence of T2DM and dyslipidaemia are estimated to be 11.2% (Li et al., 2020) and 34% (Pan et al., 2016) in China, respectively. Meanwhile, the prevalence of dyslipidaemia in patients with T2DM is estimated at 67.1%, (Yan et al., 2016), suggesting a need for a comprehensive approach to the treatment of patients with T2DM and dyslipidaemia. Statin use is the first-line approach to low-density lipoprotein cholesterol (LDL-C) level lowering and cardioprotection prescribed in addition to lifestyle interventions and glycaemic control in patients with T2DM complicated with dyslipidaemia. However, the use of a single stain may not achieve the recommended control levels or may be poorly tolerated. Meanwhile, combination treatments are not recommended because of the lack of evidence for their efficacy. (American Diabetes Association, 2020) (Jia et al., 2019). Metformin is the first-line pharmacologic treatment for T2DM (American Diabetes Association, 2019); a growing body of evidence supports its performance in antiatherosclerosis and CVD risk reduction among T2DM patients. (Petrie et al., 2020). In addition, metformin may activate adenosine 5‘-monophosphate -activated protein kinase and reduce the LDL-C levels. (Xu et al., 2015a).

Traditional Chinese Medicine (TCM) approaches reportedly improve glycaemic control, blood lipid levels, and weight management. (Wu et al., 2020). In fact, a TCM herbal formula may ameliorate blood glucose and lipid levels by shifting the composition of the gut microbiota. (Tong et al., 2018). The Jiangtang Tiaozhi (JTTZ) recipe has been used to treat patients with T2DM with dyslipidaemia for many years. (Zhao, 2013). Interestingly, the findings from a multi-centre trial suggested that it is safe and effective in the management of the blood glucose levels, lipid levels, and weight in patients with T2DM with obesity and hyperlipidaemia. (Yu et al., 2018). The formula consists of eight Chinese herbs, including Aloe [*Aloe vera* (L.) Burm. f. or *Aloe ferox* Mill. (Asphodelaceae)], *Coptidis rhizoma* [*Coptis chinensis* Franch., *Coptis teeta* Wall., or *Coptis deltoidea* C.Y.Cheng and P.K.Hsiao (Ranunculaceae)], *Anemarrhenae rhizoma* [*Anemarrhena asphodeloides* Bunge (Asparagaceae)], red yeast rice [*Fermentum Rubrum* (Aspergillaceae)], bitter melon [*Momordica charantia* L. (Cucurbitaceae)], *Salvia miltiorrhizae radix et rhizoma* [*Salvia miltiorrhiza* Bunge (Lamiaceae) ], *Schisandrae chinensis fructus* [*Schisandra chinensis* (Turcz.) Baill. (Schisandraceae)], and *Zingiberis rhizoma* [*Zingiber officinale* Roscoe (Zingiberaceae)]. In the TCM theory, the JTTZ recipe clears the excess heat in the stomach and intestines, thus alleviating patients’ symptoms. However, the mechanism underlying the clinical efficacy of the JTTZ recipe remains unknown. We hypothesised that the JTTZ may exert a therapeutic effect equal to or better than that exerted by metformin among patients with T2DM with dyslipidaemia, and that this effect may be mediated by the modulation of metabolites secreted by the gut microbiota. Therefore, a randomised, positive drug parallel-controlled, open-label clinical trial was designed; 16S rRNA gene sequencing as well as gas chromatography/time-of-flight mass spectrometry analyses will be performed to explore these mechanisms.

### Registration

The study was registered at ClinicalTrials.gov (NCT04623567) on November 5, 2020 (https://clinicaltrials.gov/ct2/show/NCT04623567).

## Methods

### Study Design

**FIGURE 1:**
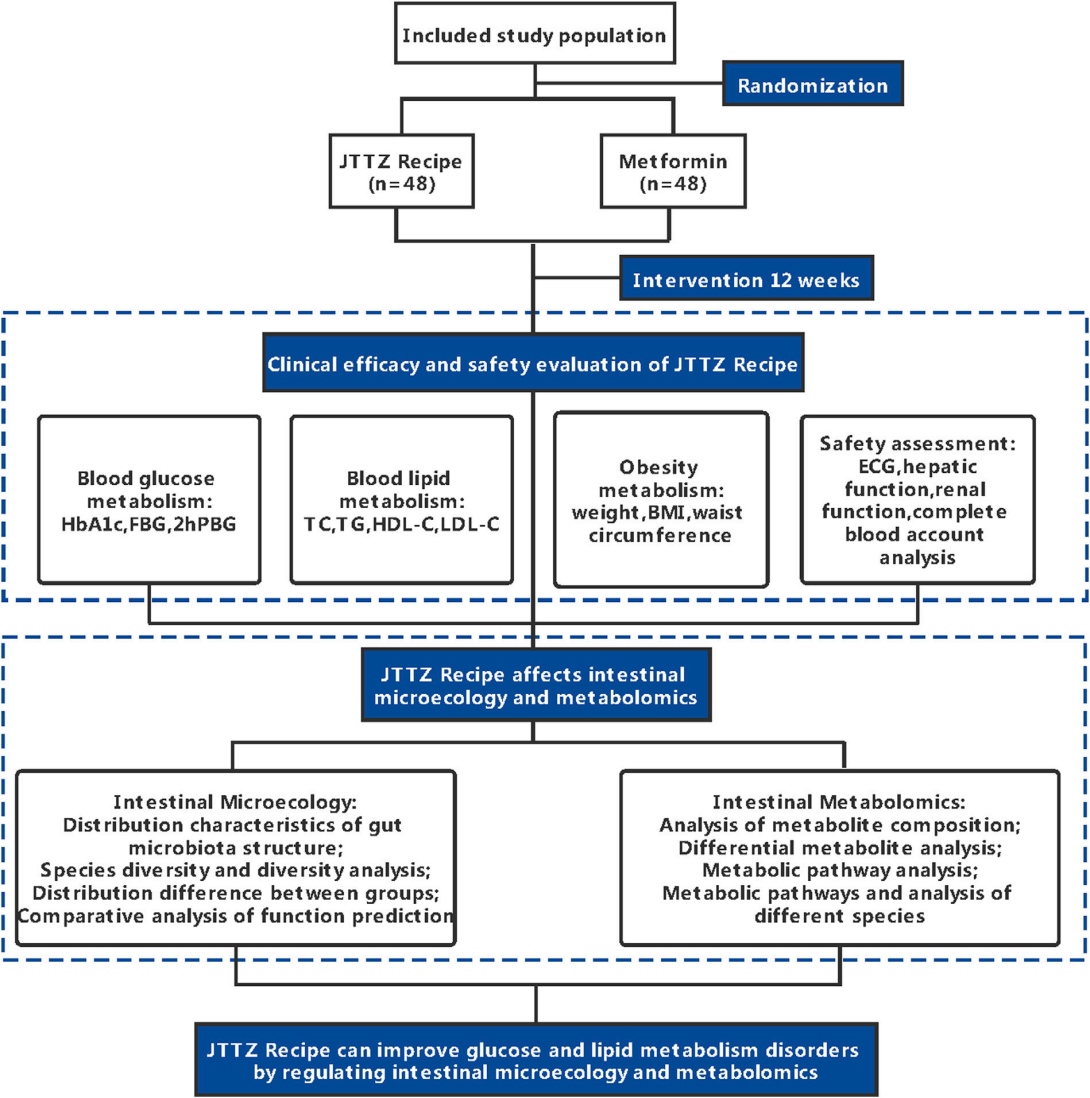
Flow diagram of the study design.

This study is a randomised, positive drug parallel-controlled, open-label, non-inferiority clinical trial in patients with glucose and lipid metabolism disturbances. In total, 96 patients diagnosed with T2DM and dyslipidaemia will be recruited for this study. The patients will be randomly divided into two groups and treated with the JTTZ recipe or metformin for 12 weeks. This study protocol follows the Standard Protocol Items: Recommendations for Interventional Trials. (Chan et al., 2013). (Supplementary File S1). The study flow is illustrated in Figure 1. All information related to the treatment schedule is presented in Table 1.

**TABLE 1:** Schedule of enrolment, assessments, and data collection.

Period	Enrolment/Baseline	Allocation	Treatment period			Follow-up period
Time points	Introduction period	0week	4 weeks	8 weeks	12 weeks	48weeks
Eligibility screening	×	—	—	—	—	—
Informed consent	×	—	—	—	—	—
Physical Examination	×	×	—	—	—	—
Characteristic	×		—	—	—	—
Random allocation	—	×	—	—	—	—
Interventions	—	—	×	×	×	—
Clinical Assessments	—	—	×	×	×	—
Medical history	×	×	×	×	×	×
Body weight	×	×	×	×	×	—
Waist circumference	×	×	×	×	×	—
Body mass index	×	×	×	×	×	—
Electrocardiogram	—	×	—	—	×	—
Routine urinalysis	—	×	—	—	×	—
Complete blood count analysis	—	×	—	—	×	—
HbA1c	—	×	—	—	×	×
FPG	×	×	×	×	×	×
2 h-PG	×	×	—	—	×	×
Blood lipid indicators	×	×	—	—	×	×
TCM symptom score	×	×	×	×	×	×
Liver and kidney function	—	×	×	×	×	—
Islet function	—	×	—	—	×	×
Urinary microalbumin	—	—	—	—	—	×
Urinary microalbumin/-creatinine ratio	—	—	—	—	—	×
Cardiac ultrasound	—	—	—	—	—	×
Carotid artery ultrasound	—	—	—	—	—	×
Biological specimens	—	×	—	—	×	—
Adverse event	—	×	×	×	×	—

FPG, fasting plasma glucose; PG, plasma glucose; TCM, traditional Chinese medicine.

### Patient Recruitment

This study aims to include 96 patients with T2DM and dyslipidaemia. Patients are recruited at the Guang’anmen Hospital, China Academy of Chinese Medical Sciences (Beijing, China). Recruitment advertisements are posted on webpages and notice boards in the hospital, presenting a brief description of the study aims, medicines and medical examinations involved, eligibility criteria, and the ways to participate in this study. Patients who fulfil the inclusion criteria and provide consent to participate in this study may be examined. Investigators will explain the study aims, requirements, and interventions to the prospective patients and ensure their understanding of the study parameters ahead of asking for their consent to participate. Patient eligibility will be confirmed by examinations performed at the endocrinology clinic by two endocrinology physicians, both of whom need to confirm patient suitability for this study. In addition, demographic characteristics, and reasons for non-participation of ineligible patients will be recorded. Recruitment began in October 2021 and will continue until July 2022. No patient was or will be involved in the design of this study, recruitment process, performing of study procedures, results reporting, or dissemination of the study findings. Physical examination, laboratory tests, and interventions will be free of charge. However, the study may not compensate its participants.

### Eligibility Criteria

#### Diagnostic Criteria of T2DM

The diagnostic criteria for T2DM are based on the *Guideline for the prevention and treatment of type 2 diabetes mellitus in China* (2020 edition), (China Diabetes Society, 2021), and include diabetic symptoms (increased thirst and urine volume, unexplained weight loss) plus any of the following: 1) random blood plasma glucose (blood glucose at any time of the day regardless of the time of last meal) levels ≥11.1 mmol/L (200 mg/dl); 2) fasting plasma glucose (FPG) level ≥7.0 mmol/L (126 mg/dl); 3) 2-h plasma glucose (2-h PG) level ≥11.1 mmol/L (200 mg/dl); and 4) glycated haemoglobin (HbA1c) level ≥6.5%.

In the absence of any symptom of diabetes, blood glucose measurements will be repeated on another day to confirm the diagnosis.

#### Diagnostic Criteria of Dyslipidaemia

The diagnostic criteria of dyslipidaemia are based on the *Chinese Guidelines on Prevention and Treatment of Dyslipidaemia in Adults* (2016, revision edition) (Chinese Joint Committee for Developing Chinese Guidelines on Prevention and Treatment of Dyslipidemia in Adults, 2016) and include any of the following: total cholesterol (TC) level ≥6.2 mmol/L (240 mg/dl), LDL-C level ≥4.1 mmol/L (160 mg/dl), high-density lipoprotein cholesterol level <1.0 mmol/L (40 mg/dl), and triglyceride (TG) level ≥2.3 mmol/L (200 mg/dl).

#### Diagnostic Criteria of TCM Syndrome

The diagnostic criteria for excess heat in the stomach and intestines syndrome are based on the *International Traditional Chinese Medicine Guideline for Diagnostic and Treatment Principles of Diabetes.* (Lian et al., 2020). The primary symptoms include abdominal distension, gastric stuffiness, and constipation. The secondary symptoms include dry mouth, bitter taste, or foul breath, thirsty and fondness for cold drinks, abnormally increased appetite with frequent hunger, red tongue with yellow coating, and rapid and forceful pulse.

The diagnosis of excess heat in the stomach and intestines syndrome is based on the presence of one main and two secondary symptoms. The TCM symptom scoring standard is presented in Supplementary File S2.

#### Inclusion Criteria

Patients are eligible for this study if they meet the following criteria: 1) age of 18–70 years at the time of enrolment; 2) diagnosis of T2DM plus 7.0 mmol/L (126 mg/dl) ≤ FPG level ≤13.9 mmol/L (250 mg/dl), or 2-h PG level ≥11.1 mmol/L (200 mg/dl) at the time of enrolment; 3) diagnosed with dyslipidaemia plus LDL-C level ≥3.4 mmol/L (130 mg/dl) and having TG level >1.7 mmol/L (150 mg/dl) at the time of enrolment; 4) have a body mass index ≥24.0 kg/m^2^ or waist circumference ≥90 cm for men and ≥80 cm for women; 5) failure to take anti-diabetic drugs regularly to reach normal blood glucose and lipid levels after diet control and exercise therapy before enrolment; 6) TCM syndrome differentiation as excess heat in the stomach and intestines syndrome; and 7) provision of signed informed consent form.

#### Exclusion Criteria

Patients are ineligible for this study if they meet any of the following criteria: 1) diagnosis of type 1 diabetes, gestational diabetes, or other special types of diabetes; 2) diabetes-related complications as the main presentation, including serious heart, lung, liver, kidney, or brain complications, or other serious primary diseases; 3) presence of diabetic ketoacidosis, hyperosmolar non-ketotic diabetic coma, severe infection, or history of surgery in the past month; 4) history of serious gastrointestinal diseases such as peptic ulcer, gastrointestinal bleeding, gastroparesis, pyloric stenosis, or gastric shunt; 5) diagnosis of psychiatric disease including alcoholism and/or psychoactive substance abuse, drug abuse, or addiction; 6) pregnancy, lactation, or intention to become pregnant; 7) history of clinical trial participation at the present or in the past month; and 8) presence of other diseases that may affect the effectiveness of study treatments or likelihood of study completion and loss to follow-up, including the unstable living or working environment, as judged by the qualifying investigator.

#### Removal, Dropout, and Termination Criteria

A patient may be removed from the study if he or she does not take study drugs as required, takes prohibited drugs (any antidiabetic drugs or TCM formulas), or refuse to cooperate with data collection procedures. Patients may withdraw from the study at any time. Included patients that fail to complete the treatment or follow-up protocols are considered as dropouts. Reasons for dropout will be recorded in case report forms (CRFs), and the last record of these patients will be included in data analysis. An investigator may decide to withdraw a patient from the study for medical reasons. The trial will be suspended in patients that experience serious adverse events (AEs) or an acute life-threatening disease. The study may be terminated at completion of randomisation masking fails or all follow-up assessments.

### Sample Size

Based on a previous study, the HbA1c level generally reduced by 1.0 and 0.9% using metformin and JTTZF for treatment, respectively. (Zhao, 2013; China Diabetes Society, 2021). The sample size was calculated by assuming a test power of 80% and a confidence level of 95%, Z_1-alpha/2_ = 1.960, alpha = 5% for the two-tailed hypothesis, Z_1-beta_ = 0.842, sigma = 0.024. A total of 38 patients are required in each group. Allowing for a 20% dropout rate during the follow-up period, the sample size was increased to at least 48 patients for each group, thus, creating a total sample of 96 patients. PASS software (version 11.0; NCSS, LLC. Kaysville, UT, United States) was used for these calculations.

### Randomisation and Allocation

The randomisation scheme was generated by the Administration Office of Drug Clinical Trial in the Guang’anmen Hospital, using the PROC PLAN procedure in SAS statistical software (version 9.4; SAS Institute, Cary, NC, United States), with a 1:1 allocation ratio to the experimental and control groups (total sample, 96 patients). The randomisation code will be released by the interactive web response system to conceal group allocation. Eligible patients will be assigned to the interventions by research assistants, according to the unique number from the randomisation scheme, the patients will receive study treatment assigned by the scheme.

### Test Drugs and Blinding

The JTTZ recipe granules used in the trial are provided by the Tianjiang Pharmaceutical Co., Ltd. (Jiangyin, Jiangsu Province, China) The metformin tablets are provided by the Guang’anmen Hospital and produced by the Shanghai Shiguibao Pharmaceutical Co., Ltd. (Shanghai, China). All drugs meet the requirements of good manufacturing practice. Although the study is open-label, the research assistants who assign patients to groups will not be aware of treatment allocation before group assignment is provided. Efficacy assessment and analysis of the results will be performed by assessors and statisticians blinded to the group allocation. All samples and data will be anonymised before analysis and evaluation.

### Interventions

Patients in the JTTZ recipe group will receive the JTTZ recipe granules (30 g per bag), one bag at a time, twice a day, to be taken with warm water after meals. Patients in the metformin group will receive 500 mg metformin tablets at a time, three times a day, to be taken with meals. The allocated interventions may be modified. In addition, this study applies the add-on therapy and the former administration of lipid-lowering agents will not be withdrawn. If AEs or voluntary withdrawal occurs, the interventions will be discontinued. Laboratory tests and drug packing return will be arranged to monitor and improve adherence.

### Outcome Measures

Demographic and clinical characteristics of the patients, including age, sex, height and weight, medical and treatment history, and examination findings, among others, will be recorded in the CRFs during a 1-week screening period and every 4 weeks during the treatment period.

### Primary Outcomes

The changes of HbA1c levels are the primary study outcomes Besides, the rates of effectively regulated HbA1c, FPG, 2 h-PG, TG, and LDL-C levels will be calculated based on the number of patients who make the target achievement. The HbA1c level ≤7%, FPG level ≤7.0 mmol/L (126 mg/dl), 2 hP G level ≤10.0 mmol/L (180 mg/dl), TG level ≤1.7 mmol/L (150 mg/dl), LDL-C level ≤3.3 mmol/L (130 mg/dl) will be set as the target achievement. The FPG levels will be tested during the screening period, before intervention, and every 4 weeks during the treatment period. The levels of HbA1c, 2 h-PG, TG, and LDL-C will be recorded during the screening period, at the beginning and at the end of the12-week treatment period.

### Secondary Outcomes

Changes to body weight, body mass index, waist circumference, and TCM symptom scores are the secondary outcomes of interest. All the parameters will be assessed during the screening period, at the beginning of the treatment period, and every 4 weeks during the treatment period.

### Biological Specimen Analysis Outcomes

The changes to the composition of intestinal microbiota, gut microbiota metabolites, and blood metabolites will be examined in faeces and blood samples at the beginning and at the end of the 12-week treatment period. The alpha-diversity, beta-diversity, principal components analysis, principal co-ordinates analysis, nonmetric multidimensional scaling, permutational multivariate analysis of variance, the linear discriminant analysis effect size of intestinal microbiota, gut microbiota metabolites, and blood metabolites will be calculated based on 16S rRNA gene sequencing and gas chromatography/time-of-flight mass spectrometry analyses. Further correlation analysis between intestinal microbiota and clinical indexes, intestinal microbiota and gut microbiota metabolites, and intestinal microbiota and blood metabolites will be performed to explore the intervention mechanism.

### Adverse Events

AEs will be continuously monitored during the 12-week treatment period and during the 36-week follow-up period. AEs indexes will include electrocardiogram findings, hepatic function tests (i.e., levels of alkaline phosphatase, aspartate aminotransferase, alanine aminotransferase, and g-glutamyl-transpeptidase), renal function tests (i.e., creatinine and urea levels), complete blood count analysis, routine urinalysis, islet function, urinary microalbumin, urinary microalbumin/creatinine ratio, cardiac ultrasound, and carotid artery ultrasound. In addition, AEs in the form of uncomfortable symptoms and other ailments will be comprehensively documented, including the timing of onset, severity, duration, treatment, and outcomes. Each AE associated with the intervention drugs will be classified as mild, moderate, or severe. Severe AEs will be submitted to the principal investigator and ethics committee within 24 h of onset. All AEs will be resolved properly. The Guang’anmen Hospital will cover non-negligent harm associated with the protocol. The AE severity evaluation criteria are as follows: 1) Mild: the patients can tolerate the discomfort, which does not interfere with the treatment, does not require special treatment or affect patient rehabilitation; 2) Moderate: the patients cannot tolerate the discomfort and require special treatment, which may affect their recovery outcomes; and 3) Severe: the patients are at risk of death or disability and require immediate emergency treatment.

### Data Management and Quality Control

To maintain high quality of the data and ensure adherence to the protocol, all investigators and research assistants were rigorously trained. The protocols for patients screening, data entry, medication use, AE reporting, and dropout recording were explained. All events will be documented on standardised CRFs. All laboratory specimens, evaluation forms, biochemical examination reports, and other records will be identified by a coded number and name only. The source medical records will be input into a professional data platform that allows for real-time data validation after being generated in each visit. The study monitoring committee (including independent data coordinators and data analysts) will perform regular data monitoring throughout the trial, review the CRFs, and ensure that information on the CRFs is consistent with that in the source medical records. The data will be independently input into the database by two research assistants. After the data are input into an electronic data platform, instant quality control and data audit procedures will be performed by the individual investigator to ensure the accuracy and reliability of these data. All study data will be managed as detailed in the full trial protocol and in accordance with the data management plan. Missing data or specific errors in the data will be sent to the investigator for a resolution. Original CRFs will be stored at the research centre for 5 years after the study completion. All the electronic data will be stored in the password-protected files on a designated computer with access restricted to the study staff. In addition, two backups will be separately stored in the Administration Office of Drug Clinical Trial in the Guang’anmen Hospital and the Clinical Evaluation Centre of the China Academy of Chinese Medical Science.

### Statistical Analysis

Intention-to-treat, per-protocol analysis, and the safety analysis will be performed. The per-protocol analysis will include only those patients who completed treatment and follow-up protocols in the allocated intervention group. Continuous variables (including age, disease duration, blood glucose, HbA1c) will be reported as means and standard deviations or as medians and interquartile ranges, as suitable. Categorical variables (including sex, past medical history, and previous treatment) will be reported as proportions. Continuous variables will be compared between the groups using analysis of variance and repeated measurement design will apply repeated-measure analysis of variance, including group and time-group interactions. The Wilcoxon rank sum test will be used to analyse non-normally distributed data. Count and grade data will be reported as frequencies (constituent ratio). Count data will be tested with the chi-square test or Fisher’s exact probability method, and grade data will be analysed using the Wilcoxon rank sum or Cochran-Mantel-Haenszel test. All statistical analyses will be performed using SPSS (version 24.0; IBM Corp., Armonk, NY, United States), and statistical significance will be set as two-sided *p*-values of ≤0.05.

### Ethics and Dissemination

This study protocol has been approved by the Ethics Committee of the Guang’anmen Hospital, China Academy of Chinese Medical Sciences (No. 2020-039-KY-01). Written informed consent will be obtained from all participants. After the trial completion, the results will be disseminated through presentations at academic conferences and publications in peer-reviewed journals.

## Discussion

This trial is designed as a randomised, positive drug parallel-controlled, open-label clinical trial. This trial will provide evidence on the effectiveness of the JTTZ recipe in regulating the blood glucose and lipid levels in patients with T2DM and dyslipidaemia. In addition, metabolomic analysis of the intestinal microorganisms may partially reveal the mechanism of the JTTZ recipe action on the intestinal microorganisms, blood glucose, and lipid metabolism.

Several studies have shown the importance of the gut microbiota in the pathophysiology of T2DM, and bacterial taxa, including the genera *Bifidobacterium, Bacteroides, Fusobacterium*, and *Blautia.* The treatment for T2DM may modulate inflammation and gut permeability, among others. (Gurung et al., 2020). A previous study showed that the intestinal flora was associated with the risk of developing T2DM, and the changes in the intestinal flora metabolites, such as short-chain fatty acid butyrate, may improve insulin response. (Sanna et al., 2019). In addition, some bacteria may contribute to the host glucose and lipid homeostasis. (Kuno et al., 2018). Our previous study demonstrated that T2DM may be alleviated by structural modulation of the gut microbiota using a traditional Chinese herbal formula, the Gegen Qinlian Decoction. (Xu et al., 2015b). The present study aims to validate the involvement of the gut microbiota in T2DM complicated with dyslipidaemia management and to identify the relevant genera of intestinal microorganisms.

This study has some limitations. First, the outcomes may be influenced by the open-label nature of this study. In addition, this is a single-centre study in China. Thus, the generalisability of any findings remains unclear.

## Abbreviations

AE, adverse event; CRF, case report form; CVD, cardiovascular diseases; FPG, fasting plasma glucose; HbA1c, glycated haemoglobin; JTTZ, Jiangtang Tiaozhi; LDL, low-density lipoprotein cholesterol; TCM, traditional Chinese medicine; TC, total cholesterol; T2DM, type 2 diabetes mellitus; TG, triglyceride.

## Abbreviations

AE, adverse event; CRF, case report form; CVD, cardiovascular diseases; FPG, fasting plasma glucose; HbA1c, glycated haemoglobin; JTTZ, Jiangtang Tiaozhi; LDL, low-density lipoprotein cholesterol; TCM, traditional Chinese medicine; TC, total cholesterol; T2DM, type 2 diabetes mellitus; TG, triglyceride.
